# Effects of Moisture Absorption on the Mechanical and Fatigue Properties of Natural Fiber Composites: A Review

**DOI:** 10.3390/polym17141996

**Published:** 2025-07-21

**Authors:** Ana Pavlovic, Lorenzo Valzania, Giangiacomo Minak

**Affiliations:** Department of Industrial Engineering, University of Bologna; Via Luciano Montaspro, 97, 47121 Forlì, Italy; lorenzo.valzania@studio.unibo.it (L.V.); giangiacomo.minak@unibo.it (G.M.)

**Keywords:** natural composites, mechanical properties, water absorption, degradation, fatigue

## Abstract

This review critically examines the effects of moisture absorption on the mechanical and fatigue properties of natural fiber composites (NFCs), with a focus on tensile strength, elastic modulus, and long-term durability. Moisture uptake can cause reductions in tensile strength of up to 40% and in elastic modulus by 20–30% depending on fiber type, mass fraction (typically in the range of 30–60%), and surface treatments. The review highlights Ithat while surface modifications (e.g., alkaline and silane treatments) significantly mitigate moisture-induced degradation, their effectiveness is highly sensitive to the processing conditions. Additionally, hybridization strategies and optimized fiber orientations show promise in enhancing fatigue resistance under humid environments. Despite substantial progress, major challenges remain, including the lack of standardized testing protocols and the limited understanding of multiscale aging mechanisms. Future research directions include developing predictive models that couple moisture diffusion and mechanical deterioration, implementing advanced in situ monitoring of damage evolution, and exploring novel bio-based treatments. By addressing these gaps, NFCs can become more reliable and widely adopted as sustainable alternatives in structural applications.

## 1. Introduction

Natural fiber composites (NFCs) are increasingly recognized as promising alternatives to synthetic fiber reinforced composites offering advantages such as low cost, biodegradability, and reduced environmental impact. Typically composed of plant-derived fibers (e.g., flax, hemp, and jute) embedded in polymeric matrices, these materials are gaining traction in various engineering sectors, including automotive, construction, and packaging. Their appeal lies in the growing demand for sustainable materials that align with circular economy goals and reduce dependence on fossil-based resources.

The operational properties of natural fiber composites are primarily governed by the fiber mass fraction, the chemical composition of the fibers (including cellulose, hemicellulose, and lignin content), and the type of surface treatments applied. In general, the mass fraction of natural fibers in composites ranges from 30 to 60% depending on the application and desired properties. Typical fiber contents are around 40–50% for flax and hemp, and slightly lower, about 30–40% for jute composites. Higher fiber fractions generally improve stiffness and strength but may increase moisture sensitivity, whereas chemical modifications (e.g., alkali or silane treatments) enhance interfacial bonding and reduce water uptake. In addition, special reinforcements such as nanocrystalline cellulose can significantly enhance mechanical performance and barrier properties when properly dispersed and integrated.

Despite these benefits, the mechanical and fatigue properties of NFCs still lag behind those of glass or carbon fiber composites. Moisture absorption remains a critical challenge: due to their hydrophilic nature, plant fibers readily absorb water, compromising the fiber–matrix interface, reducing load transfer efficiency, and accelerating damage mechanisms such as swelling, delamination, and microcracking. These effects not only lower stiffness and strength but also significantly impair fatigue resistance—one of the most critical performance criteria for structural applications.

Over the past decade, an expanding body of research has addressed specific aspects of NFCs’ behavior, including tensile properties, environmental effects, aging, and surface treatments. However, most studies have focused on isolated variables or single failure modes. As a result, there is a lack of comprehensive understanding of how moisture absorption influences both mechanical and fatigue performance under various conditions, including temperature, fiber orientation, treatment type, and fiber volume fraction.

Recent reviews have also emphasized the importance of hygrothermal effects and surface modifications on NFCs’ performance [1].

This review aims to bridge that gap by systematically analyzing the effects of moisture absorption on the mechanical and fatigue properties of natural fiber composites. The scope is limited to plant-based fibers in polymer matrices with particular attention to the interaction between environmental exposure and structural performance. The review also examines the role of surface treatments (e.g., alkaline and silane) in mitigating degradation, compares dry and wet fatigue behavior, and discusses experimental data from multiple studies under a wide range of loading and aging conditions.

By providing an integrated perspective across mechanical, environmental, and durability domains, this work intends to serve as a reference for researchers and engineers seeking to improve the performance and reliability of bio-based composites in real-world applications.

## 2. Mechanical Properties

The mechanical properties of plant fiber composites are generally inferior to their synthetic counterparts with tensile strength values up to 10 times higher for carbon fiber reinforced composites. However, values reported in the literature for natural composites are close to those of glass fiber composites. In a 2012 study by Genc et al. [2], tensile tests were performed on polyester matrix composites reinforced with either glass or flax fiber. The Young’s modulus of the glass fiber composite was measured at 15.1 GPa compared to 10.2 GPa for the flax fiber composite. Tensile strength was approximately 1.7 times higher for synthetic composites.

Xu et al. (2023) demonstrated that higher fiber content and optimized orientations significantly affect moisture diffusion and mechanical properties, confirming the importance of controlling these parameters [3].

Additionally, a meta-analysis conducted by Blanchard and Sobey in 2019 [4] highlights that, in the literature, glass fiber composites generally outperform flax fiber composites. Table 1 provides a comparison of glass- and flax-based composites in terms of elastic modulus and tensile strength. However, the results for plant fibers show significant variability, suggesting considerable potential for improvement through further studies aimed at optimizing pre-treatment processes or composite fabrication techniques. As can be seen, the elastic modulus of natural composites is 38% lower while the tensile strength is about 1/4 of that of glass composites. However, surface treatments and hybridization with synthetic fibers can partially fill the performance gap.

Shubhra et al. [7] report that natural composites can achieve values up to 1.2 times lower than those of glass fiber composites. Various solutions can be employed to bridge this performance gap. The most common approaches include applying surface pre-treatments to the fibers or hybridizing plant fibers with higher-performing ones such as carbon fibers.

To provide additional guidance and practical context, common testing standards (e.g., ASTM D3039 for tensile tests and ASTM D7264 for flexural tests) and the corresponding types of equipment typically used in the literature are given in Table 1. This information supports reproducibility and helps future researchers quickly identify suitable methods and instrumentation.

### Hybrid Composites and Improvement of Mechanical Properties

In 2013, Ramnath et al. [8] and Zivkovic et al. [9] conducted a study to evaluate the effectiveness of hybrid composites through tensile, flexural, and impact tests.

The laminates were fabricated manually, with GFRP (Glass Fiber Reinforced Polymer) layers applied to both outer surfaces, impregnated with epoxy resin, and cured under pressure for 3–4 h. The inner layers, composed of jute and/or abaca fibers arranged alternately in orthogonal directions, were impregnated with a resin and hardener mixture, achieving an overall fiber content of 40% by volume.

The tests, conducted at room temperature and 40% relative humidity, examined three composites: jute-GFRP, abaca-GFRP, and abaca-jute-GFRP. Tensile tests produced stress–strain curves that were similar across all composites. The mechanical properties are summarized in Table 1, which shows that jute and abaca composites exhibit high tensile strength but a lower elastic modulus compared to the individual fibers. The modulus was calculated based on the linear portion of the stress–strain curves.

The data also reveal that the percentage elongation of hybrid composites with dual natural fibers (abaca-jute-GFRP) is higher than that of single-fiber composites, indicating greater deformation capacity before failure and enhanced ductility.

Including jute fiber in hybrid composites significantly improves strength more than any other natural fiber, as shown by Plackett et al. in 2003 [10].

In the literature, hybrid composites are generally made using two main techniques:

1. Inter-ply, which involves stacking layers of different fiber fabrics into a single laminate;
2. Intra-ply, which uses co-woven fabrics with alternating threads of dissimilar fibers.

Zhang et al. [11] demonstrated that in inter-ply glass/carbon hybrid composites, the stacking sequence does not affect tensile strength but significantly influences flexural properties.

A detailed comparison between inter-ply and intra-ply configurations was conducted by Md Zahirul Islam et al. [12], testing carbon/flax hybrid laminates. In the inter-ply laminate, single layers of carbon fiber fabric (1C) and double layers of flax fiber fabric (2F) were alternated following a 1C-2F-1C-2F-1C stacking sequence. In contrast, the intra-ply laminate featured woven fabric alternating carbon fibers (49%) and flax fibers (51%). The polymer matrix was a low-viscosity epoxy resin mixed with a chemical agent to promote cross-linking.

The tensile test results showed superior performance for the inter-ply laminates with a maximum stress σ_MAX_ = (805.26 ± 26.58) MPa and Young’s modulus E = 46.61 GPa compared to the intra-ply laminates (σ_MAX_ = (765.46 ± 8.47) MPa, E = 36.94 GPa), despite the greater thickness of the intra-ply laminates (3.1 mm vs. 2.8 mm). Both laminates exhibited the same elongation at break (~3.8%) but the intra-ply laminates displayed less marked fluctuations before Ifailure. This was attributed to the higher flax fiber content in the intra-ply configuration and their greater homogeneity, which provided improved damping properties.

An interesting feature observed was the bilinear behavior before final failure as documented by Derbali et al. [13] and with monotonic tensile tests as documented by Pio Michele Giuliani et al. [14] confirming the transition value found in the literature at 0.2% of strain. This composite behavior can be explained, in fact, by two main phases:

1. Initial phase (higher slope): The matrix and fibers work in synergy, with the matrix uniformly transferring the load to the fibers, resulting in high stiffness.
2. Post-transition phase (lower slope): The matrix begins to microcrack or deform plastically, reducing its load transfer capacity. As a result, the fibers bear a greater proportion of the load, leading to an overall reduction in stiffness.

This behavior reflects the fibers’ and matrices’ combined mechanical properties, demonstrating a gradual transition from the initial phase to the subsequent one.

## 3. Fatigue Resistance

Fatigue in materials is a degradation phenomenon that occurs under cyclic or variable loads, potentially leading to sudden structural failure even under stresses below the material’s elastic limit. Responsible for approximately 90% of failures in mechanical components, fatigue is particularly significant in the aerospace, civil engineering, and automotive sectors.

The fatigue limit is the stress value below which a material does not experience fatigue failure, regardless of the number of applied cycles. However, this threshold may not exist in more ductile composites as even at very low stresses the material can fail if subjected to a sufficiently high number of load cycles. This behavior is observed, for instance, in aluminum and, in certain cases, in natural composites where plant fibers do not exhibit high performance.

Recent work by Jandas et al. (2024) provided a comprehensive analysis of fatigue behavior under humid environments, supporting our observations on moisture-induced fatigue degradation [15]. The use of composite materials has increased, particularly in applications requiring high strength-to-weight ratios. Compared to metallic materials, composite materials exhibit fatigue behavior influenced by several factors:

Fiber orientation: Fatigue resistance is not maximized in composites with fibers entirely aligned with the applied load as they exhibit poor transverse resistance. Configurations with fibers at 90° (cross-ply) or angled at 5–10° relative to the primary axis improve performance, as demonstrated by tests on glass fiber composites [16].

Matrix type: Matrices with higher fatigue resistance, such as epoxy resin, perform better due to their intrinsic hardness and resistance to deterioration.

Fiber volume fraction: A higher fiber content increases fatigue resistance, provided the fibers are evenly distributed. Increases in fiber volume fraction (Vf) enhance the S–N curve. However, an optimal value exists, around 70% for glass fibers, beyond which performance tends to stabilize or deteriorate [16].

Type of loading: Unlike metals, composites exhibit different resistances depending on the type of loading (e.g., shear or tension) and the load ratio (R).

Regarding the load ratio R, composites display unique behaviors in stress–strain graphs particularly concerning the hysteresis loop area. This area, proportional to energy dissipation during loading, varies significantly with the number of cycles and the R-value:

For R = 0.1 (tension–tension), the area of the hysteresis loop progressively decreases as the number of load cycles increases. Initial cycles show larger areas, supporting the hypothesis of high damage activity during the initial stages, as observed by Fuwa et al. [17]. They noted, in fact, a peak in acoustic emission, indicative of energy dissipation within the first 200 cycles for epoxy resin composites reinforced with carbon fibers, followed by a saturation of emission after approximately 1000 cycles.

Towo and Arnold [18], in a study on sisal fiber composites, confirm this trend: beyond 1000 cycles, a reduction in the hysteresis loop area is observed, consistent with Fuwa et al.’s analysis of fatigue behavior.

For R = −1 (tension–compression), the hysteresis loop area exhibits an opposite trend to R = 0.1. In unidirectional (UD) composites, which are weaker under compressive loads than tensile loads, the initial hysteresis curves are narrow, indicating minimal damage. However, as the number of cycles increases, the area of the curves grows progressively, reflecting the propagation of microcracks and plasticization of the matrix, resulting in energy loss.

Experimental studies on fiber reinforced composites show that fatigue damage typically begins with the formation of cracks in the plies whose fibers are oriented at the widest angle relative to the load direction. In cross-ply laminates, for example, cracks initially develop in the plies oriented at 90° (perpendicular to the load) and propagate across the entire load-bearing section of the ply. This phenomenon can occur even after short periods of use with loads lower than 20% of the laminate’s static tensile strength.

**Figure 1:**
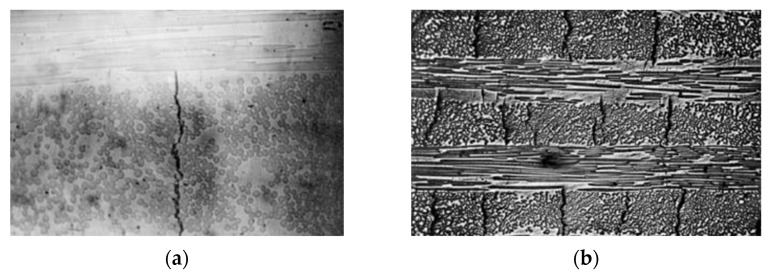
Simple crack (**a**) and multiple cracks (**b**) in composite cross-ply laminates [16].

However, these cracks do not extend to adjacent plies if those fibers are sufficiently aligned with the load direction, as shown in Figure 1, where vertical cracks stop once they reach a ply with horizontal fibers [13].

When the load exceeds 25–30% of the laminate’s static strength, the accumulation of fatigue cycles leads to the formation of delamination cracks near the interfaces between plies. These cracks progressively develop, reducing the overall stiffness and increasing the likelihood of failure in the weakest or most heavily loaded ply. The remaining life of the laminate at this stage represents less than 10% of the component’s total life.

Fatigue cracks may also appear in plies with fibers parallel to the load in the final stages of life. These cracks originate from the breakage of a fiber or the matrix itself, causing debonding if the fiber–matrix adhesion is insufficient.

In high Young’s modulus composites (e.g., carbon fiber, boron, and Kevlar), the transverse fatigue strength is comparable to that of glass fiber composites. Still, the longitudinal strength is significantly higher due to their low strain-to-failure and resistance to environmental agents. These materials exhibit an almost constant tensile strength close to the static value, up to 10^7^ load cycles.

### 3.1. Influence of Fiber Fraction

Shah et al. [19] analyzed the fatigue behavior of J190/polyester composites manufactured with jute fibers (190 twists/meter) and fiber percentages ranging from 17% to 38%. In quasi-static tests, tensile strength increases proportionally with fiber content, in agreement with the rule of mixtures, with an excellent coefficient of determination (R^2^ = 0.974), confirming the validity of the theoretical model. This indicates that natural fiber composites follow standard micromechanical models when manufactured using fibers from the same batch.

The data strongly correlates with S-N curves (R^2^ > 0.95) in tension–tension fatigue tests, again highlighting predictable behavior consistent with theoretical models. Polymer Fiber Composites (PFCs) with higher fiber percentages exhibit better static properties and greater fatigue resistance, like carbon fiber composites. The material’s fatigue strength coefficient (b), related to the slope of the curves in the S-N plot, remains stable across all fiber percentages, indicating that the rates of fatigue strength degradation are similar regardless of fiber volume.

A lower (b) value implies a steeper S–N curve slope, with increasing cycle numbers, indicating faster fatigue strength degradation. The coefficient (b) is expressed in Equation (1), derived from Paris’ law for fatigue crack growth:

(1) $$S_{MAX}=S_{0}N^{b}$$

Increasing the fiber percentage in a PFC improves both static and fatigue performance. However, beyond an optimal fiber percentage (>40–45%), several studies [20,21,22,23] show that fatigue performance deteriorates. This is due to poor fiber distribution during manufacturing, which increases regions with high fiber volume fractions and fiber/matrix interfaces. While these interfaces facilitate stress transfer, they also become points where microcracks form and propagate.

Mandell et al. [23] demonstrated that a fiber content exceeding 40–45% reduces the (b) coefficient, worsening fatigue resistance despite improvements in static properties.

In the case of J190/polyester composites, increasing the fiber percentage alters the failure mechanism in fatigue tests. At low fiber volumes, matrix cracks form perpendicular to the load, leading to brittle failure with smooth fracture surfaces. At higher fiber percentages, fewer matrix cracks form, but failure becomes more catastrophic with more severe fractures, irregular fracture surfaces, delamination, and longitudinal splits.

In a further study by Abdul Hakim Abdullah et al. [24], the fatigue behavior (stress ratio: R = 0.5) of an epoxy resin composite reinforced with Kenaf fiber, pre-treated with 6% NaOH, was analyzed for fiber volume percentages of 15% and 45%.

The UTS values for composites with different Kenaf fiber volume percentages, compared to pure resin, are reported in Table 2.

Fatigue behavior is analyzed for variable stress levels (σ_MAX_): 90%, 80%, 70%, 60%, and 50% of the UTS. The results are summarized in Table 2, where the fatigue behavior of epoxy resin with Vf = 15% and Vf = 45% is included for reference. These findings confirm the results described in Shah et al. [19].

The fatigue life of natural fiber reinforced composites follows the previously described trend. However, as the fiber percentage in the composite increases, the fatigue strength coefficient (b) is reduced. This corresponds to steeper slopes, indicating faster fatigue strength degradation of the material. 

However, the fatigue stress limits for a given number of load cycles remain consistently higher for composites with a more significant reinforcement percentage. Nevertheless, depending on the type of fiber, this trend may not always hold, with a possible overlap of S–N curves between composites with higher and lower fiber percentages. This overlap indicates that there may be a threshold number of cycles beyond which the fatigue life of the composite with a lower fiber percentage becomes superior.

### 3.2. Influence of Fiber Orientation

Shah et al. [19] in 2013 fabricated polyester specimens reinforced with flax fibers, testing various fiber orientations: UD ([0°]_4_ and [90°]_4_) and biaxial ([±45°]_4_). In static tests, laminates with fibers oriented at 0° to the loading axis exhibited significantly superior performance with values 11 times and 3 times higher than laminates with fibers oriented at 90° and ±45°, respectively.

However, since plant fibers are highly anisotropic (and consequently, so are the composites derived from them), it has been demonstrated that [±45] laminates provide better responses compared to unidirectional (UD) laminates when the applied load is angled by at least 30° relative to the main axis [25]. This scenario is common in practical applications where axial loading cannot always be assumed.

**Figure 2:**
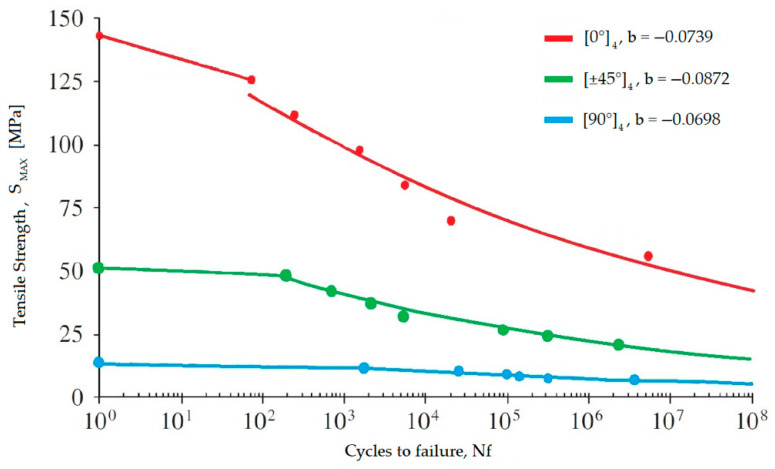
S–N diagram: Fatigue life data for F50/polyester composites with different textile architectures [19].

For axial loads, as shown in Figure 2 and previously observed for synthetic fibers, the fiber orientation misaligned with the loading direction (45° or 90°) leads to a significant reduction in the composite’s static UTS, which translates to a lower fatigue load-carrying capacity over the entire cycle life.

Encouragingly, the degradation rate for biaxial laminated natural composites (with fibers at 45°) appears better than that of unidirectional, biaxial, and triaxial glass fiber composites. This is due to the higher fatigue resistance coefficient and significantly better low-cycle fatigue properties of PFCs compared to GFRPs, as indicated by the position of the curves on the graph, where higher curves suggest less degradation with an increasing number of load cycles.

**Figure 3:**
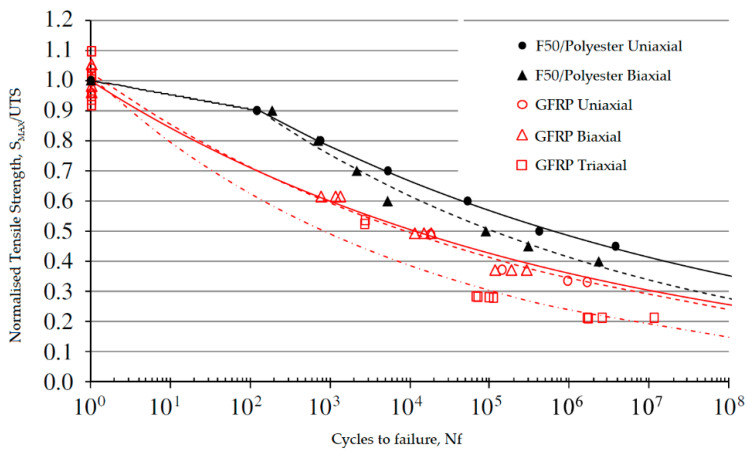
Normalized S–N diagram: Tensile-tensile fatigue performance of uniaxial and multi-axial composites reinforced with flax fibers and E-glass [19].

As seen in Figure 3, the triaxial GFRP exhibits the worst fatigue strength degradation rate, as evidenced by the slope of the S–N curve and its lower position on the S/UTS–N graph. This is attributed to a failure mechanism distinct from uniaxial and biaxial composites where matrix failure begins with the detachment of the ±45° layers and then delamination from the 0° material [23].

On the other hand, UD composites fail under axial loads (0°) due to fiber/matrix debonding, whereas under off-axis loads (90°), failure occurs by merging cracks along the interfaces.

In 2014, Liang et al. [26] analyzed the fatigue behavior of epoxy resin and flax fiber laminates, reporting the following *UTS* values in quasi-static tensile tests:

[0°]_12_: *UTS* = 318 MPa
[90°]_12_: *UTS* = 26.1 MPa
[0/90°]3S: *UTS* = 170 MPa
[±45°]3S: *UTS* = 79 MPa

A higher number of fibers aligned at smaller angles to the loading axis corresponds to a higher UTS.

It is observed that samples with fibers oriented at ±45° have superior fatigue resistance compared to others under the same normalized load, reaching the threshold at 0.5*UTS*, compared to 0.4*UTS* for 90° orientation and <0.4*UTS* for other configurations. This data reflecting the fatigue life degradation rate confirms the superiority of specimens with fibers at 45°.

In absolute terms, the S–N graph shows that composites with fibers parallel to the loading axis exhibit higher breaking stress values and a worse fatigue strength degradation rate than those with angled orientations, particularly at 45°.

### 3.3. Hybridization for Improving Fatigue Life

Natural and synthetic fibers can be hybridized within a common polymer matrix to enhance the mechanical properties of natural fiber reinforced composites and reduce environmental impact. This technique combines the desirable properties of both fiber types while mitigating their weaknesses. For instance, despite their excellent tensile strength, carbon fibers exhibit low toughness, which can be improved by combining them with flax fibers. This also enhances the impact resistance of carbon fiber reinforced composites [27].

Hybridization can be achieved through layered fabrics with different fibers (inter-ply composites) or co-woven fabrics with alternating threads of different fibers (intra-ply composites) [12]. In quasi-static tensile tests, inter-ply laminates showed a maximum stress *σ_MAX_* of 805.26 ± 26.58 MPa and Young’s modulus *E* = 46.61 GPa, while intra-ply laminates recorded a *σ_MAX_* of 765.46 ± 8.47 MPa and E = 36.94 GPa. However, fatigue tests conducted under oscillating loads at 70% of the breaking stress and a frequency of 3 Hz revealed that intra-ply laminates exhibited an almost 2000% higher fatigue life than inter-ply laminates despite their lower maximum stress. Intra-ply composites reached nearly 550,000 cycles before failure compared to 20,000 cycles for inter-ply composites. This result is confirmed by the superior internal cohesion of intra-ply composites post-failure.

When compared to single-fiber composites (flax-only or carbon-only), intra-ply hybridization increases fatigue life by 1000% compared to flax-only composites. Conversely, inter-ply composites showed lower fatigue life than flax-only composites, likely due to variations in manufacturing processes or fiber quality.

On the other hand, carbon fiber composites were woven triaxially. They achieved a fatigue life of approximately 1,000,000 cycles, which could have been closer to the intra-ply configuration with a different architecture [12].

Under cyclic loading, failure begins in fibers with lower strain-to-failure, meaning the carbon fibers break before flax fibers.

In inter-ply hybrids, the breakage of carbon fibers creates a continuous path for crack propagation. In contrast, in intra-ply composites, flax fibers interspersed between carbon fibers interrupt crack propagation, distributing micro-damage uniformly. This mechanism explains the superior fatigue resistance of intra-ply composites, which exhibited significantly longer fatigue life than inter-ply composites. In situ SEM micrograph analyses could provide deeper insights into the failure mechanisms.

**Figure 4:**
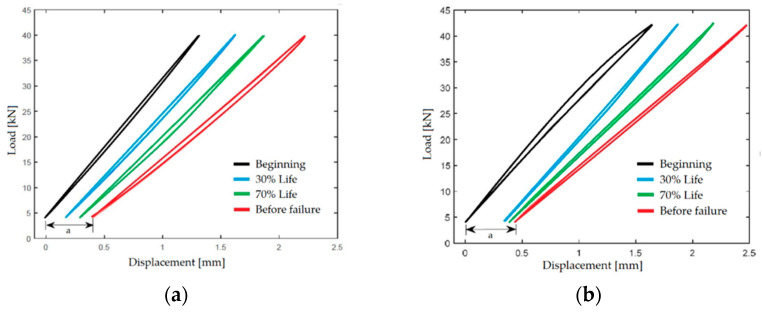
Hysteresis cycle at the beginning of the test, at 30% fatigue life, 70% fatigue life, and before failure for hybrid composites: (**a**) inter-ply and (**b**) intra-ply.

Hysteresis cycle analysis during fatigue tests indicates that intra-ply composites with a lifespan of 547,227 cycles dissipate less energy per cycle than inter-ply composites, which only achieve 12,739 cycles, as shown in Figure 4.

The greater closure of hysteresis loops in intra-ply composites indicates lower energy dissipation. In contrast, the downward shift in the lower part of the hysteresis loop represents plastic deformation caused by cyclic micro-fractures. Interestingly, this deformation is similar in both configurations despite intra-ply composites exhibiting an approximately 2000% greater fatigue life.

The energy dissipated per cycle (*E_d_*) is calculated as the difference between the total energy used for deformation (*E_p_*) and recovered energy. To monitor the progression of damage in materials, the loss factor (*η*) is used, which measures the proportion of energy dissipated relative to the potential energy in a fatigue cycle.

(2) $$\eta =\frac{E_{d}}{2\pi E_{p}}$$

This parameter is sensitive to variations in material stiffness and, as discussed earlier, is correlated with Young’s modulus, which tends to decrease as fatigue life progresses.

## 4. Surface Treatments

This section analyzes potential solutions to enhance the mechanical performance of natural composites.

Physical modifications to natural fibers improve the mechanical adhesion between fiber and matrix by acting on surface properties without altering chemical ones. Physical treatments, such as corona, plasma, UV, beating, and thermal treatments, optimize the fiber–matrix interface. Wu et al. (2010) [28] observed that rougher fiber surfaces promote better wettability and greater contact with the matrix, reducing fiber–matrix debonding.

Chemical modifications, on the other hand, enhance fiber–matrix adhesion through chemical reactions.

A 2015 study conducted by Tran et al. [29] demonstrated that surface treatments increase the surface energy of composite components, thus improving inter-adhesion. Surface energy can be estimated by measuring the contact angle between a liquid droplet (distilled water) and the surface, using Young’s equation in the Kaelble–Owens–Wendt method [30]. Lower contact angles indicate higher surface energy, confirming the effectiveness of treatments; however, ensuring accurate results requires rigorous, highly specific testing, and might be inaccurate if not conducted properly.

Advances in fiber surface modifications, including the use of novel coupling agents and silane treatments, have been recently summarized by Hosseini et al. (2023), highlighting improvements in both water resistance and mechanical strength [31].

The surface treatments presented and analyzed below, concerning improving mechanical properties, fall under chemical treatments. These treatments are more cost-effective in terms of materials and equipment than physical treatments, making them much more widely used.

### 4.1. Alkaline Treatment in Enhancing Mechanical Properties

Alkaline treatment, a simple, cost-effective, and efficient method, is one of the most widely used techniques for modifying the cellulosic structure of natural fibers using a sodium hydroxide (NaOH) solution. This treatment primarily breaks hydrogen bonds in the fiber’s network structure, significantly increasing surface roughness and removing hemicellulose, lignin, waxes, and oils from the outer cell wall. This process also leads to cellulose depolymerization.

At optimal concentrations, alkaline treatment not only cleans the fiber, removing lignin and hemicellulose, but also realigns fibers into robust semi-crystalline structures and splits them into thinner fibrils increasing its specific surface area and reducing surface defects, thereby improving mechanical properties, as demonstrated by Griffith in 1920. This enhances adhesion between the hydrophilic fiber and the hydrophobic matrix.

However, the correct alkali concentration is crucial, as excessive dosage can cause surface peeling and compromise fiber properties, as shown in multiple studies. A 2020 study by Siakeng et al. [32] on PLA/PALF/CF composites (PLA, pineapple fibers, and coconut fibers) reported significant increases in tensile strength with a 112.3% improvement for the highest percentage of coconut fibers and a 136.55% improvement for an equal distribution of coconut and pineapple fibers.

**Figure 5:**
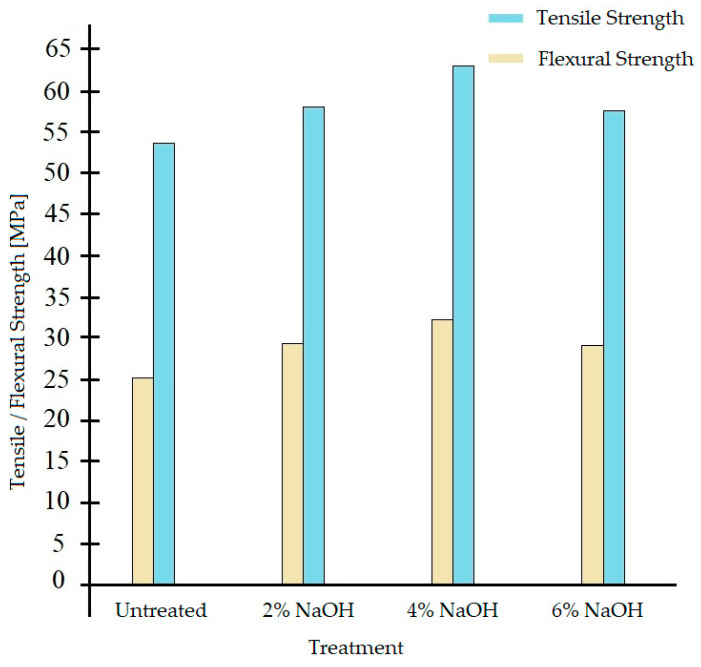
Tensile and flexural strength of a propylene composite reinforced with untreated hemp fibers and after treatment with 2%, 4%, and 6% alkali [33].

**Figure 6:**
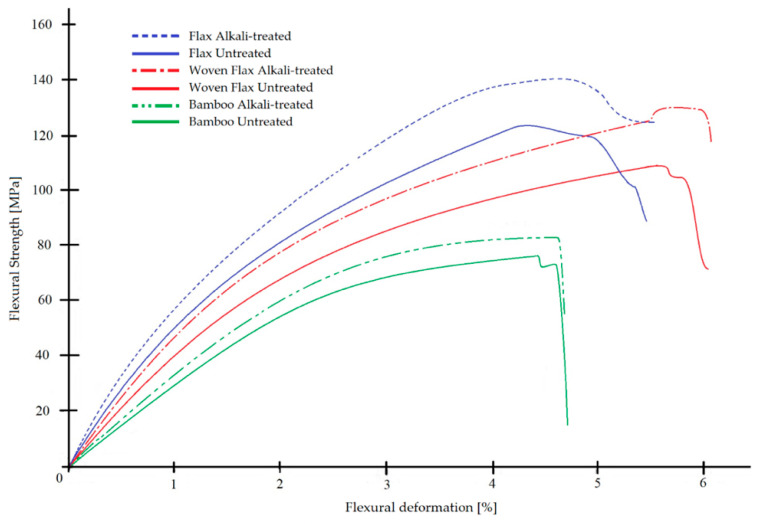
Increased mechanical bending properties for bamboo and flax fiber composites after alkaline treatment [34].

Figure 5 [33] and Figure 6 [34] show enhanced mechanical properties for treated samples with peak tensile and flexural strength observed at a NaOH concentration of 4%. Beyond this concentration, performance decreases.

In 2022, Seisa et al. [35] compiled the best results from various studies regarding surface treatments including alkali treatment and silane treatment as a coupling agent. Silane is a molecule with both a hydrophilic and a hydrophobic end capable of acting as a bridge between the polymer matrix and the natural fiber, enhancing adhesion, load transfer, and mechanical properties. The results, noteworthy for their significance, are presented in Table 3.

### 4.2. Alkali Treatment in Fatigue Life Improving

Among surface treatments, the most widely used is alkaline treatment due to its ease of application, low cost, and significant benefits, particularly for static properties when used in appropriate concentrations.

Regarding fatigue resistance, a 2007 study by Towo and Ansell [18] analyzed the impact of alkaline treatment on sisal fiber bundles in polyester and epoxy resin matrices. The study highlighted significant improvements in the mechanical properties and fatigue resistance of treated composites compared to untreated ones, with more pronounced advantages for polyester matrix composites.

For composites with a polyester matrix, the average tensile strength was measured at 223 MPa for untreated fibers, whereas treated fibers (0.06 M NaOH) exhibited an improved tensile strength of 286 MPa. Similarly, untreated fibers showed a tensile strength of 329 MPa for composites with an epoxy resin matrix, which increased to 334 MPa after alkaline treatment with 0.06 M NaOH.

Fatigue data show that alkaline treatment ensures greater durability of composites, especially under low cyclic loads, specifically the following:

- Table 4 reports the results for polyester and epoxy matrix composites tested at different stress levels and with a load ratio of R = 0.1 (tension–tension).
- Table 5 presents data from tension–compression tests (R = −1) conducted on laminates with epoxy resin matrix.

An analysis of Table 4 and Table 5 might suggest that treated composites exhibit poorer fatigue performance than untreated ones. However, this is due to the reference to the applied maximum stress percentage. Thanks to the improvement in static properties and the increase in ultimate tensile strength (UTS), treated composites can withstand higher loads.

**Figure 7:**
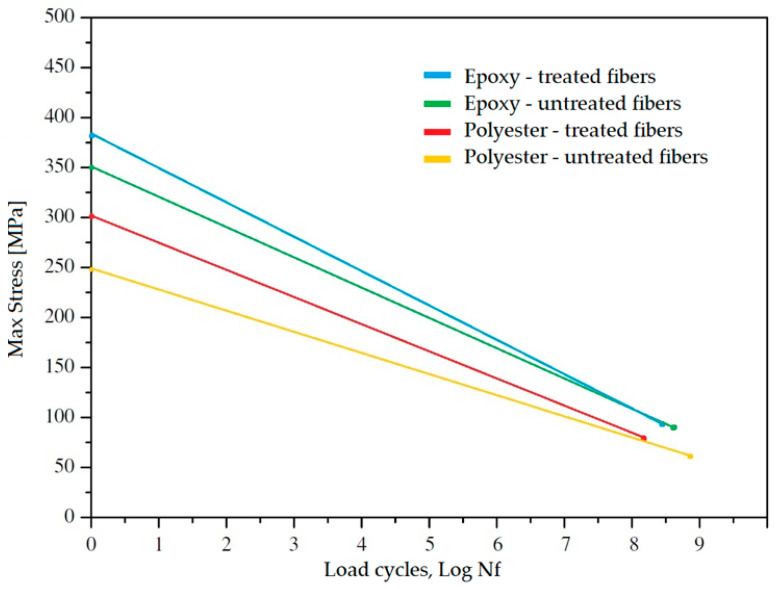
Stress–life cycle regression lines for untreated and NaOH-treated polyester/epoxy fiber composites at R = 0.1 (includes static strengths of composites).

At the same unnormalized stress level, treated composites show a significant increase in cycles to failure with R = 0.1 and R = −1, as graphically illustrated in Figure 7.

Although alkaline treatment increases the rate of degradation of fatigue resistance (greater negative slope of the curve), the overall improvement in static performance translates into an increase in the fatigue life of the composites.

## 5. Moisture Absorption

Polymeric composites made with natural fibers can absorb moisture through water immersion or exposure to a humid environment. This phenomenon compromises the interaction between natural fibers (NFRs) and the polymer matrix (PM), reducing stress transfer efficiency and negatively affecting the material’s physical, mechanical, and thermal properties. These effects are intensified by surface roughness, fiber swelling, and delamination.

Natural fibers are particularly susceptible to moisture due to polar functional groups, such as hydroxyl (-OH) groups found in cellulose, hemicellulose, and lignin, which form hydrogen bonds with water molecules. Moisture can initially be retained through surface adsorption (without penetration) and then diffuse deeper via true absorption.

Surface adsorption can be classified into physisorption (weak interactions like van der Waals forces, reversible at low temperatures) and chemisorption (strong chemical bonds, often irreversible and occurring at high temperatures).

Water absorption (*W_A_*) is the primary mechanism responsible for water retention in natural composites. Under exposure to humid environmental conditions, the literature reports *W_A_* values ranging from 0.7 to 2% after 24 h, 1–5% after one week, and up to 18–22% after several months [36]. The formula used to calculate water absorption is the following:

(3) $$W_{A}(\%)=\frac{W_{f}-W_{0}}{W_{0}}\cdot 100$$

where *W_f_* is the composite’s final weight after immersion, and *W_o_* is its initial weight.

Among the components of natural fibers, hemicellulose is mainly responsible for moisture absorption. Fibers with a high hemicellulose content exhibit greater absorption, leading to swelling, fiber–matrix debonding, mechanical property loss, and increased microbial attacks, accelerating the biodegradation of fibers and composites.

A study conducted by Wang et al. in 2006 [37] found that moisture absorption in composites affects their electrical conductivity. While no current flow was initially observed, once approximately 50% of the saturation moisture level was absorbed, electrical conductivity increased with water content due to the role of water molecules in the gaps between the fibers and the matrix.

This finding could help measure moisture absorption when weighing the sample is not feasible. However, caution is required in applications involving current, as excessive moisture could alter its flow.

The hollow cavities of natural fibers, while reducing composite density, increase their absorption capacity, affecting properties such as crystallinity, strength, and swelling.

At room temperature, moisture absorption generally follows Fick’s Law, reaching saturation relatively quickly, with free water molecules flowing from macro- and micro-voids at a linear rate, gradually slowing until saturation.

However, retained moisture increases significantly at higher temperatures, showing non-Fickian behavior with variable diffusivity and prolonged absorption over several months. This phenomenon, known as dual-phase absorption, might be explained by the increase in hydrogen bonding between water molecules and hydrophilic polymer chains or by the presence of interstitial voids amplified by water’s plasticizing effect [38].

Water transport in composites can occur through capillarity along the fiber–matrix interface, diffusion into the less hydrophobic matrix, or defects such as pores and microcracks. Initial absorption causes swelling in the cellulose fibers, generating interfacial stresses that lead to microcracking in the matrix, further facilitating moisture ingress. Once saturation is reached, the accumulated water may cause the release of water-soluble chemicals from the fibers, resulting in a definitive fiber–matrix debonding through the formation of osmotic pockets and interfacial voids, as documented for unsaturated polyester composites reinforced with hemp fibers [39].

Studies on glassy polymers (below their glass transition temperature, Tg) reveal that moisture transport occurs through concentration-driven diffusion and polymer matrix relaxation. This is observable in a two-stage diffusion (bi-linear non-Fickian), with an initial rapid Fickian phase followed by a slower second phase. The latter is associated with the reorganization of polymer chains in the presence of the liquid penetrant (in this case, water), creating new intermolecular spaces for further absorption [40].

**Figure 8:**
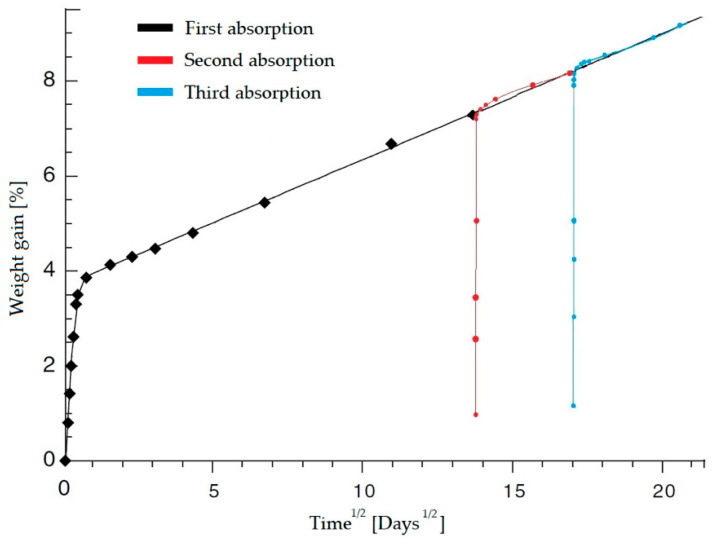
Reabsorption phases: Gain in weight % of moisture with respect to time. The reabsorption phases begin at the end of the previous one (desorption time is not considered).

In reabsorption cycles, after a desorption phase under controlled conditions, an increased absorption capacity is observed compared to the initial cycle. This phenomenon is attributed to the permanent plasticization of the polymer, which generates new intermolecular voids. The linear (Fickian) initial reabsorption phase reaches the maximum moisture level of the previous phase, surpassing the original linear limit, followed by a second non-Fickian phase, which further alters the polymer structure. This behavior can be seen in Figure 8.

A post-absorption–desorption thermal treatment near Tg can restore the reabsorption curve to its initial behavior, demonstrating that the structural changes induced by moisture are physical in nature and reversible [41].

### 5.1. Moisture Absorption in Relation to Fiber Content

Moisture absorption in composites varies significantly depending on the type and percentage of reinforcement. A recent study by OGAH et al. [42] analyzed the moisture absorption of three natural fillers in an epoxy matrix: wood flour, pumpkin stem flour, and rice husk. Although the fillers are particulate rather than fibrous, the results are also indicative of fibrous composites, showing differences in saturation times:

Rice husk: The composite with 30% by weight of rice husk exhibited the lowest moisture absorption (16.5%) and minimal thickness swelling (0.35%), indicating low hydrophilicity, ideal for applications in humid environments.

Wood flour: At 30% by weight, the composite achieved a moisture absorption of 35.3% and swelling of 1.62%, the highest among the three materials, indicating higher hydrophilicity than rice husk.

Pumpkin flour: With 30% by weight, this filler resulted in the highest absorption (40.5%) but with swelling (1.4%) lower than wood flour.

Diffusion coefficients increase with the filler percentage, confirming that the chemical composition of the reinforcement directly affects absorption.

An additional study by Tajvidi et al. [43] on polypropylene matrix composites with various reinforcements (wood flour, rice husk, kenaf fibers, and newspaper fibers) found that at a 25% reinforcement level, moisture absorption remained limited, with maximum values at around 2% for newspaper fiber. In contrast, at 50%, composites with kenaf and newspaper fibers showed a significant increase with absorption reaching up to 13%.

The observed non-Fickian behavior indicates that for high fiber contents the saturation process requires extended periods, highlighting the need for further studies for long-term applications in humid environments.

The moisture absorption behavior in composites varies significantly with the type of reinforcement and matrix. After five weeks of immersion, absorbed moisture ranged from a minimum of 4.76% (rice husk) to a maximum of 13.19% (kenaf fiber). These results emphasize the importance of selecting reinforcements based on the intended application.

The matrix also plays a crucial role in influencing maximum absorption, especially at high reinforcement percentages. Variations in matrix-reinforcement adhesion and hydrophilicity contribute to these differences.

Composites with hemp fiber and polyethylene matrices (HDPE and LDPE) show increased absorption with higher fiber content. However, differences of up to 3.5 percentage points are observed between the two matrices for the same fiber content, highlighting the impact of matrix density on absorption behavior.

### 5.2. Degradation of Mechanical Properties Post-Moisture Absorption

Exposure to humid environments or prolonged immersion significantly degrades the mechanical properties of composites, particularly those reinforced with natural fibers, which are more hydrophilic than synthetic fibers. For synthetic composites, moisture absorption reaches approximately 1% of the weight at saturation compared to values exceeding 40% for natural composites, making the latter critical for study.

A test conducted by Panthapulakkal et al. at the University of Toronto [44], following ASTM D570-22 standards [45], analyzed samples with various compositions of polypropylene (PP), glass fibers, hemp fibers, and compatibilizing agents, maintaining a constant 40% fiber volume fraction. After 3624 h of immersion in distilled water, tensile tests revealed significant mechanical losses in composites with natural fibers: up to a 40% reduction in tensile strength and a 60% reduction in Young’s modulus compared to unaged specimens.

For pure PP, limited moisture absorption and its hydrophobic nature preserved almost all mechanical properties.

Samples with a high percentage of hemp fiber exhibited marked deterioration, particularly in the elastic modulus E. Even glass fibers, though less hydrophilic, did not prevent long-term degradation. These effects can be attributed to the extended immersion period, often overlooked in other studies.

A 2023 study by Abdela et al. [46] examined natural composites with a PLA (polylactic acid) matrix that is biocompatible and biodegradable. The matrix, made more ductile with a plasticizer (6% vol.), demonstrated lower moisture absorption without compromising mechanical properties. PLA-Enset fiber composites, with fiber content ranging from 15% to 25% Wf, exhibited significant losses in tensile properties post-immersion, confirming the vulnerability of natural composites in humid environments.

After moisture absorption, composites show a substantial reduction in mechanical properties. For samples with high fiber and low plasticizer content, tensile strength decreases by 16.7% while Young’s modulus decreases by 20%. The pure matrix, enhanced with the plasticizer, shows lower losses than the polypropylene analyzed earlier, highlighting its potential for bio-sustainability.

The flexural properties, however, remain almost unchanged after absorption, increasing with the fiber percentage to the same extent as they degrade due to moisture absorption. The post-immersion flexural strength converges to similar values (~73.5 MPa) regardless of the fiber percentage, allowing for potential economic savings and process optimization.

Enset-PLA composites, although experiencing slight degradation, perform comparably to other PLA biocomposites and PLA–linen composites [47]. After aging, stiffness improves with increased fiber content, with a maximum elastic modulus of 5.33 GPa (dry fibers) and 4.24 GPa (aged fibers) for the 25% fiber sample. The results are presented in Table 6.

Environmental humidity also affects mechanical properties. A 2022 study by Pantaloni et al. [47] on flax–PLA composites (40% vol. fibers) demonstrated that water absorption is proportional to relative humidity (RH). At 50% RH, the composite absorbs ~2.77% water, while at 75% RH, it reaches ~3.55%, compared to ~8.76% (98% RH) and 15.12% in immersion. Fick’s law adequately describes the absorption process, correlating absorbed moisture with the degradation of mechanical properties, which intensifies with the level of saturation.

The quasi-static test data highlight a drastic deterioration of both elastic modulus and tensile strength for specimens exposed to very high humidity (98% RH) and immersion. At the same time, no significant differences were observed between 50% RH and 75% RH. The main values are as follows:

- Young’s modulus (GPa): from 7.4 ± 0.6 to 4.9 ± 0.4 (immersion);
- UTS (MPa): from 54.3 ± 1.6 to 32.3 ± 1.3 (immersion);
- Strain at failure (%): increase from 1.5 ± 0.1 to 1.8 ± 0.3 (immersion).

The increase in strain at failure is attributed to matrix plasticization caused by water absorption.

These results align with the 2019 study by Habibi et al. [48], which analyzed flax–epoxy resin composites saturated by immersion at 45 °C and tested at different operating temperatures (23 °C, 50 °C, and 75 °C). The study confirms that moisture absorption causes a reduction in tensile strength and Young’s modulus, as well as an increase in strain at failure.

### 5.3. Influence of Post-Desorption on the Mechanical Properties of Composites

Investigating the effect of moisture absorption up to saturation (Wet) and the subsequent desorption process (Wet/Dry) on composites’ static and fatigue behavior is essential. Mejri et al. in 2018 [49] studied HDPE samples reinforced with short birch fibers (SBF, 40% by volume) subjected to quasi-static flexural tests after different aging conditions: Ambient conditions and aging by immersion at 60 °C (to accelerate moisture absorption) with tests performed both in the wet state (Wet) and after desorption (Wet/Dry).

Moisture in Wet samples significantly reduces mechanical properties: flexural modulus decreased by 47.24%, the maximum stress was reduced by 50.81%, and maximum strain increased by 58.07% compared to unaged samples due to the plasticizing effect of water.

In contrast, Wet/Dry samples nearly fully recovered their mechanical properties, reaching the flexural modulus that is 98.82% of the original value (2.51 GPa), for maximum stress 96.28% (54.10 MPa), and for strain at failure 96.45% (5.98%).

These results indicate that desorption allows for almost complete recovery of mechanical properties, although moisture absorption causes residual plastic deformations. While these reductions are modest, they can accumulate during repeated absorption–desorption cycles, leading to drastic reductions in mechanical properties.

### 5.4. Effects of Surface Treatments on Absorption Properties

Pre-treatment of natural fibers in composites improves mechanical properties and reduces surface hydrophilicity, enhancing fiber–matrix adhesion and minimizing water absorption. Reduced absorption ensures better performance in humid or immersed environments.

#### Effects of Coupling Agents on Moisture Absorption

A 2003 study by Botros and Chemicals [50] showed that untreated wood fiber composites experience swelling up to 400 times greater than treated ones.

Aggarwal et al. in 2015 [51] analyzed HDPE composites with wood fiber and bamboo flour at various reinforcement levels (10–50% by weight), treated with maleated polyethylene (MAPE) as a coupling agent. Relative to adsorption, tests under relative humidity conditions (33–90%) at 30 °C revealed that untreated composites exhibited weight gains 1.15–1.4 times higher (wood fiber) and 1.1–1.2 times higher (bamboo flour) than treated ones.

Considering absorption, instead, after 600 h of immersion in distilled water at 30 °C, samples with 10% treated fibers absorbed 0.5–0.75% moisture, compared to 0.65–0.97% in untreated ones. With 50% reinforcement, absorption was 3.5–3.6% (treated) and 6.1–4.62% (untreated).

The average reductions in absorption due to treatment ranged from 23% to 62.1% (wood fiber) and from 10.7% to 56.2% (bamboo flour), confirming the effectiveness of coupling agents in limiting absorption and preserving mechanical properties.

Silane (SiH_4_) is a coupling agent that interacts with OH-rich surfaces of natural fibers and the polymer matrix, enhancing adhesion and reducing water absorption. A study by Tokushima University (2019) [52] on PLA/SBF composites (short bamboo fibers) compared to the untreated (absorption of 18.8% after 250 h of immersion), post-alkaline treated (absorption of 22.4%, indicating fiber damage probably due to the very high level of NaOH % in the solution), and silane-treated (reduced absorption of 17.6% due to the strengthening effect of the treatment) samples.

Coupling agents (including silane) demonstrate a significant ability to reduce moisture absorption in composites, protecting mechanical properties and improving durability in challenging environments. These findings suggest that water absorption decreases with silane treatment (over one percentage point lower than untreated composites), preventing partial water molecule penetration and improving interfacial adhesion between the PLA matrix and bamboo fibers.

### 5.5. Alkaline Treatment and Moisture Absorption

Alkaline treatment, one of the most common processes for modifying natural fibers, improves mechanical properties and fiber–matrix adhesion in composites. It involves immersing fibers in sodium hydroxide (NaOH) solutions at optimal concentrations (4–5%), removing lignin, wax, and surface oils, increasing fiber roughness, and reducing hydrophilic hydroxyl groups. However, excessively high concentrations can weaken the fibers by removing lignin and hemicellulose (that is, on the one hand, they are primarily responsible for water retention, but on the other hand, they are also responsible for structural strength).

The treatment also improves the quality of fiber–matrix adhesion, as fibers become thinner with increased specific surface area, reducing surface defects and interstitial voids. This results in a significant reduction in moisture absorption.

A study by Siakeng et al. in 2020 [32] examined hybrid natural composites made of a PLA matrix (70%), coconut fibers (CF), and pineapple leaf fibers (PALF) at 30%. Samples treated with a 6% NaOH solution were compared to untreated ones, using various fiber ratios: C1P1 (15% CF + 15% PALF), C3P7 (9% CF + 21% PALF), and C7P3 (21% CF + 9% PALF).

After immersion in distilled water for seven days, it was found that samples with a higher percentage of CF absorbed less moisture due to the low cellulose content in coconut fibers. Alkaline-treated samples showed significantly reduced absorption compared to untreated ones, attributable to the removal of hydroxyl groups and improved fiber–matrix adhesion.

Alkaline treatment reduces moisture absorption, but excessively high NaOH concentrations may have the opposite effect. The literature studies highlight that optimal concentrations minimize Water Absorption (WA) while treatments with 10% NaOH or higher degrade the surface of natural fibers, increasing absorption.

Gudayu et al. (2020) [53] compared untreated sisal fibers with fibers treated with 10% NaOH and exposed for four hours at 65% RH. The treated ones showed a saturation WA of 20.68% by weight (vs. 18.57% for untreated fibers) and a diffusion coefficient increasing from 2.26 × 10^−11^ to 2.32 × 10^−11^ m^2^/s.

This confirms that high NaOH concentrations enhance moisture absorption, making them unsuitable for humid environments or immersion composites.

A post-treatment with hydrogen peroxide (H_2_O_2_) can mitigate the adverse effects of alkaline treatment, neutralizing alkaline residues and improving fiber–matrix adhesion.

Rajesh et al. in 2018 [54] analyzed fibers treated with up to 15% NaOH followed by H_2_O_2_, finding a significant reduction in WA even for high percentages of NaOH%: fibers treated with NaOH 5% + H_2_O_2_ achieved 6.59% WA, whereas NaOH 10% + H_2_O_2_ only reached 5.73% WA and NaOH 15% + H_2_O_2_ achieved 4.60% WA.

These results indicate that post-treatment can enhance performance even at high alkali concentrations. However, further studies are needed to compare optimal alkaline treatments with those followed by H_2_O_2_, evaluating their impact on moisture absorption, mechanical properties, and fatigue resistance.

## 6. Effects of Temperature on Moisture Absorption

Temperature significantly affects the performance of composite materials, causing degradation at high temperatures and brittleness at low temperatures. This study examines three temperature ranges: high (>70 °C), moderate (25–70 °C), and ambient/subzero (≤30 °C).

### 6.1. High Temperatures (>70 °C)

Exposure temperature influences the mechanical properties and moisture absorption of composites. The literature documents weight loss due to thermal degradation, which can be divided into three main phases [55]:

First Phase (≈50 °C–≈100 °C):

Polymers’ glass transition temperature (Tg) is often reached during this phase, approximately 60 °C for polyester and between 70 °C and 167 °C for epoxy resin [56]. Structural deformations and stiffness loss occur. Moisture release through desorption may cause up to 10% weight loss, but this phase might not occur if the retained moisture content is low.

Second Phase (≈210 °C–≈350 °C):

This phase involves reaching the melting temperature of the matrix, which is 150–162 °C for PLA and 250–300 °C for polyester [56]. Stiffness in the composite is compromised. Hemicellulose degradation occurs between 213 °C and 238 °C, and lignin degrades at higher temperatures, resulting in a mass loss of approximately 60% [55].

Third Phase (≈350 °C–≈800 °C):

Thermal degradation of natural fibers is completed in this phase. Residual lignin and cellulose are eliminated, leaving less than 20% of the initial mass at the final thermal stability range of 650–800 °C [55].

The second phase is absent for fibers treated with NaOH because lignin and hemicellulose have already been removed. However, degradation in the third phase begins at lower temperatures than untreated fibers [57].

**Figure 9:**
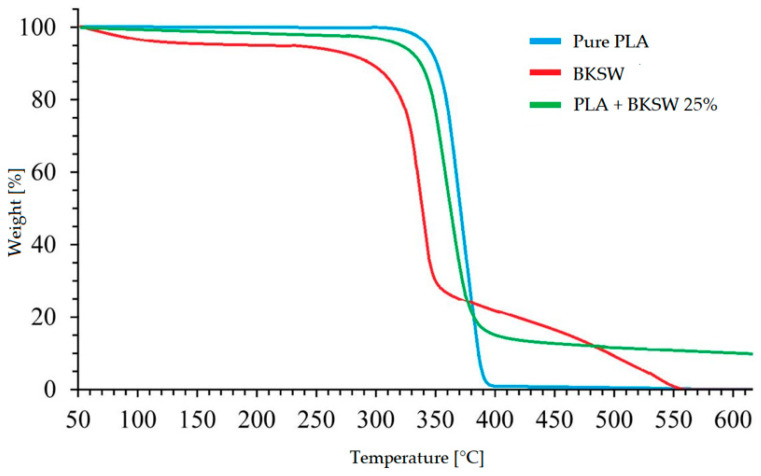
Thermal degradation and percentage weight reduction for PLA, bleached wood fibers (BKSW), and their composite, at 25% fiber weight [58].

These phases, evident in the slope changes in thermal curves (Figure 9), illustrate how natural composites entirely lose their structural capacity at high temperatures, making it challenging to quantify the mechanical loss after surpassing the glass transition temperature.

### 6.2. Moderate Temperatures (25–70 °C)

An increase in temperature within this range raises the absorption rate and the maximum saturation limit of composites, particularly natural plant-based ones, known for the high hydrophilicity of their fibers. This phenomenon is valid until critical temperatures are reached, beyond which rapid material degradation prevents further analysis.

A study conducted by Dip Saikia in 2010 [59] examined the water absorption of natural fibers such as Sanseveria (bowstring hemp), okra, and betel nut, immersed in distilled water at temperatures between 27 °C and 67 °C (300–340 K). Fibers with higher cellulose content demonstrated higher absorption.

**Figure 10:**
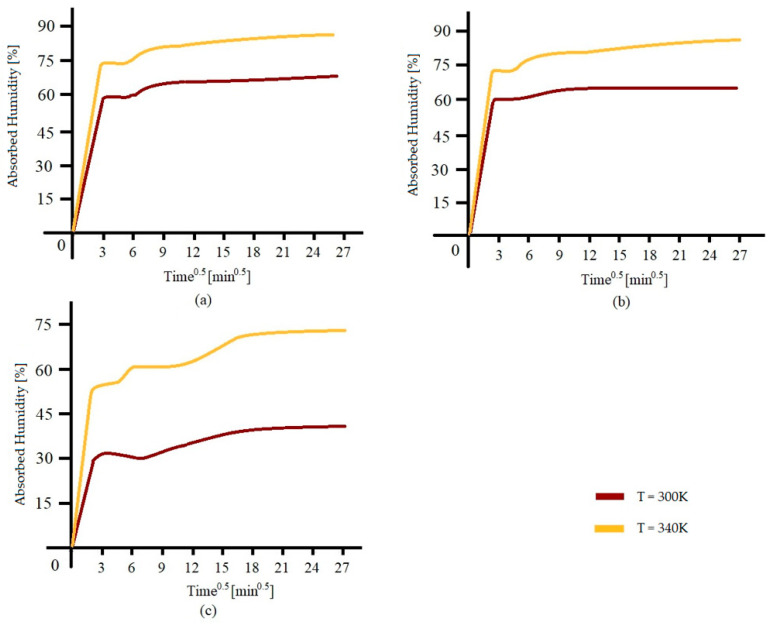
Weight % of water absorbed by (**a**) bow hemp, (**b**) okra, and (**c**) betel nut fibers as a function of time at different temperatures [59].

The gravimetric curves in Figure 10a–c show an initial Fickian behavior, reaching 60% of the total absorption, followed by a nonlinear (Non-Fickian) behavior until saturation.

Okra fibers (76% cellulose) exhibited greater absorption than betel nut fibers (44% cellulose), consistent with the role of cellulose as the primary hydrophilic component. In contrast, lignin, being less hydrophilic, negatively influences absorption.

As the temperature increases, there is an observed rise in both the absorbable water and the maximum water content at saturation, explained by increased molecular mobility and reduced liquid viscosity. Table 7 confirms this relationship with rising values of absorbed water content and diffusion coefficients as a function of water temperature and cellulose percentage.

A 2006 study conducted by Retegi et al. [60] analyzed polypropylene samples reinforced with flax pulp (2–15% by weight) immersed in water at 30, 50, 70, and 100 °C. Diffusion coefficients increase with temperature for both fibrous reinforcements and particulate fillers (e.g., with coupling agents MAPP1 and MAPP2, as shown in Table 8), confirming that higher temperatures facilitate moisture absorption regardless of the type of reinforcement.

### 6.3. Ambient/Subzero Temperatures (t < ~30 °C) with Variations in Seawater

Studies on composites reinforced with plant fibers at low temperatures and in extreme environments, such as saltwater (marine environments) and subzero conditions, show that moisture absorption predominantly follows Fick’s diffusion law. “Green” composites, a relatively recent innovation, remain under research and development, with limited yet promising studies.

A 2010 study by Deo et al. [61] analyzed composites with an epoxy resin matrix reinforced with Lantana Camara fibers, an invasive plant used to create reinforcements. Samples (10%, 20%, 30%, and 40% by weight of fiber) were exposed to three conditions for 80 h: saturated vapor (100% RH) at room temperature, immersion in saltwater at room temperature (marine environment), and subzero exposure (~−10 °C, RH 90–100%).

Tests were conducted on three samples for each condition and fiber percentage. Moisture absorption increased with fiber content, reaching its peak at 40% by weight of reinforcement. This was attributed to interstitial voids, poor fiber–matrix adhesion, and the high hydrophilicity of Lantana Camara, which is rich in cellulose and free OH groups that form hydrogen bonds with moisture.

Regarding saturation times and absorption levels, the samples exposed to vapor reached it in ~60 h with 13.72% WA by weight, the ones immersed in the marine environment in ~70 h with 8.90% WA by weight, and the ones exposed to a subzero environment in ~70 h with 2.30% WA by weight.

The absorption process is more gradual in marine and subzero environments compared to vapor.

Higher temperatures facilitate greater absorption, although the comparison between vapor and marine environments yielded unexpected results, with vapor showing higher absorption than immersion, contrary to expectations.

The study did not analyze immersion in distilled water but focused on the marine environment, i.e., saltwater. In these conditions, NaCl ions on the surface of immersed fibers may initially delay water diffusion, reducing the absorption rate. This behavior aligns with previous research on glass fiber reinforced composites, which observed reduced initial absorption in marine environments. However, prolonged exposures (over 18 months) revealed significant surface degradation compared to immersion in distilled water, leading to loss of mechanical properties and a progressive increase in moisture absorption.

In subzero conditions, moisture absorption was significantly lower than in other environments. This phenomenon can be attributed to the reduced mobility of water molecules at low temperatures and the diminished formation of intermolecular hydrogen bonds between fibers and water despite high surrounding humidity.

The recorded absorption curves follow the Fickian model, and the diffusivity coefficient *D*, representing the ability of water molecules to penetrate the composites, which was calculated using Boltzmann’s relation for thin laminates:

(4) $$D=\pi {\left[\frac{h}{4M_{\infty }}\right]}^{2}{\left[\frac{M_{2}-M_{1}}{\sqrt{t_{2}}-\sqrt{t_{1}}}\right]}^{2}$$

where *M_∞_* indicates the saturation moisture content, *h* is the composite laminate thickness, and *t*_1_ and *t*_2_ are two time points in the linear absorption interval with *M*_1_ and *M*_2_ as the corresponding moisture content values [62].

The data indicate an increase in the saturation moisture content *M_∞_* and the diffusivity coefficient with rising fiber content, regardless of the environment. This behavior was most pronounced in vapor-exposed samples, intermediate for those immersed in saltwater, and minimal for those in subzero conditions.

At the end of environmental aging, the tensile strength of the composites was compared to the initial dry condition: results showed an increasing trend in tensile strength up to 30% fiber content, followed by a decline at higher percentages. In the vapor environment, where maximum diffusivity and saturation absorption were recorded, there was a drastic reduction in mechanical performance with tensile strength decreasing by up to 65% compared to the dry sample for higher fiber percentages.

Conversely, in subzero conditions characterized by minimal diffusivity and absorption, the loss in tensile strength was less pronounced, making this the most favorable condition for preserving mechanical properties.

In addition to tensile strength, flexural strength was analyzed. Here, too, the maximum strength increase was observed at 30% fiber content, regardless of the exposure environment. The degradation of properties followed a similar trend to tensile strength: composites absorbing higher percentages of moisture at saturation exhibited more significant performance reductions, with subzero conditions providing the best results.

### 6.4. Distilled Water/Marine Environment/Subzero Conditions

In 2019, the Department of Mechanical Engineering in Orissa published a study [63] to expand on the results obtained nine years earlier. The research compared epoxy resin composites reinforced with Luffa Cylindrica fibers under various environmental conditions: immersion in distilled water, saltwater (5% NaCl), and subzero conditions (−25 °C). The samples were fabricated using a hand lay-up method with single (SL), double (DL), and triple layers (TL) of fibers (62% cellulose, 20% hemicellulose, and 11.2% lignin) with a maximum fiber content of 20% by weight to maintain optimal proportions.

The specimens were aged until moisture saturation, with weight increments measured every 12 h using Equation (1). Saturation was considered achieved when variations between successive measurements were below 0.1%. The saturation times varied depending on the environment: 84 h for distilled water, 120 h for saltwater, and 108 h for subzero conditions. Moisture uptake increased with the number of layers: 17.23% (salt water), 19.00% (distilled water), and 2.30% (subzero) for triple-layer specimens, highlighting an increase in free OH groups with higher fiber content, as observed in studies on composites reinforced with L. Camara fibers [62].

Subzero conditions showed again the lowest moisture absorption, followed by saltwater, where NaCl ions deposited on fiber surfaces seemed to impede water diffusion. Finally, distilled water resulted in the highest absorption levels. This reduction in moisture uptake is significant as it implies less degradation of mechanical properties. The maximum equilibrium moisture content (EMC) values are as follows:

- Saltwater: min(SL) = 8.05% and max(TL) = 17.23%;
- Distilled water: min(SL) = 9.05% and max(TL) = 19.00%;
- Subzero (−25 °C): min(SL) = 1.46% and max(TL) = 2.13%.

The absorption process followed Fickian behavior, which was consistent with the moderate temperatures in the study. Diffusivity coefficients (D) were calculated using Equation (2). As expected, D increased with fiber volume content and was highest for composites immersed in distilled water, lower for saltwater, and lowest under subzero conditions. The D values were comparable to those of jute and sisal composites in polyester or epoxy matrices reported in the literature [64]. However, determination coefficients (R^2^) indicated greater reliability for saltwater conditions, while subzero conditions exhibited lower accuracy.

Post-aging mechanical properties, both tensile and flexural strengths, confirmed previous findings: increased moisture uptake led to more significant degradation of composite mechanical properties.

- Tensile strength: Properties improved with increasing fiber content up to an optimal level (below 30% by volume) beyond which ultimate tensile strength (UTS) decreased. Higher moisture absorption in distilled water compared to saltwater and subzero conditions exacerbated UTS reduction, particularly for composites with higher fiber content due to greater hydrophilicity. In distilled water, tensile strength losses compared to dry specimens were 17% for SL, 26% for DL, and 31% for TL.
- Flexural strength: Similar trends were observed, with maximum reductions of 14.28% (SL), 19% (DL), and 22.2% (TL) in distilled water. An unusual result was found for TL composites exposed to subzero temperatures, showing a 3.7% increase compared to unaged specimens. This anomaly was attributed to slight fiber expansion due to minimal moisture uptake, which filled interstitial voids and enhanced fiber–matrix adhesion. This behavior was also noted by Ayensu [65] in 2000 for polymer composites reinforced with jute fibers.

These findings also apply to hybrid composites, as Pai et al. [66] demonstrated in a 2022 study on aramid and basalt fiber composites with epoxy resin matrices. While basalt fibers are less hydrophilic than natural fibers, their higher moisture absorption than synthetic fibers still results in mechanical property degradation. After saturation (180 h), tensile, flexural, and impact strengths were measured under three conditions: immersion in distilled water (25 °C), exposure to vapor (60% RH, 40 °C), and subzero temperatures (−25 °C). Mechanical property retention percentages correlated with moisture absorption levels with more significant degradation associated with higher moisture uptake. However, subzero samples exhibited reduced degradation due to less plasticization from moisture, given the lower mobility of water molecules at low temperatures.

Moisture absorption percentages were 5.44% for immersion in distilled water at 25 °C, 3.12% for exposure to −25 °C, and 1.80% for exposure to 60% RH, 40 °C.

These results confirm that moisture absorption remains a critical factor in the degradation of mechanical properties for non-hybrid and hybrid composites.

### 6.5. Influence of Freeze/Thaw Cycles on Moisture Absorption and Mechanical Properties

Natural fiber reinforced composites exhibit reduced mechanical degradation and limited moisture absorption at subzero temperatures, making them suitable for such environmental conditions. Additionally, pre-applied fiber treatments, such as silane treatments, can further enhance their mechanical strength and hydrophobicity. However, it is crucial to investigate the effects of potential freeze/thaw cycles in environments with annual thermal fluctuations, which may lead to the progressive deterioration of composites.

A 2023 study by Balan et al. [67] analyzed epoxy resin composites reinforced with flax fibers and palm seed powder filler. The results showed that freeze (−20 °C) and thaw cycles caused consistent degradation of mechanical properties (tensile strength, flexural strength, and hardness) with a more rapid decline after 30 cycles. During freezing, absorbed water can freeze even at the fiber–matrix interface, leading to expansions that create microcracks and increase the material’s capillarity. During thawing, the space created by expansion facilitates further moisture absorption, intensifying interstitial damage and the diffusion coefficient.

The previous results confirmed from [68,69,70,71,72] on cementitious specimens showed how moisture absorption and the diffusion coefficient increase with freeze/thaw cycles. While these findings can also be extended to hydrophilic materials like natural composites, specific studies certifying this behavior in natural fiber composites are lacking, highlighting the need for further research.

## 7. Influence of Moisture Absorption on Fatigue Resistance

Moisture absorption in composite materials, especially those reinforced with plant fibers, is closely linked to the degradation of mechanical properties due to the high hydrophilicity of the fibers. The effects of aging caused by freeze–thaw cycles, leading to degradation in tensile, flexural, and impact strengths, have been discussed. This section focuses on analyzing the impact of moisture on the fatigue life of plant-based composites.

Studies show that when immersed or exposed to moisture, plant-based composites absorb more water than other materials. This increased absorption causes plasticization of the polymer matrix, fiber damage, and crack formation in the matrix. These phenomena negatively affect fatigue performance, resulting in a decrease in Young’s modulus and an increase in strain at failure.

A comparison between Dry and Wet samples reveals that the Wet ones exhibit lower maximum stresses and higher failure strains. In Wöhler (S–N) diagrams, this translates into a downward curve shift, with lower initial values for low cycle counts. However, for low applied load levels relative to UTS, an increase in fatigue life is observed in samples aged due to moisture absorption compared to unaged ones. This behavior is attributed to the plasticization of the matrix, which increases residual strain at the end of each cycle, allowing the material to endure loads for a more extended period before failure.

Habibi et al. in 2019 [48] compared unaged flax–epoxy composite samples with samples saturated by immersion at 45 °C. These were subjected to quasi-static tensile tests and fatigue tests at 23 °C, 50 °C, and 75 °C with maximum loads of 0.8, 0.7, 0.6, and 0.5 UTS at a frequency of 5 Hz and with R = 0.1.

**Figure 11:**
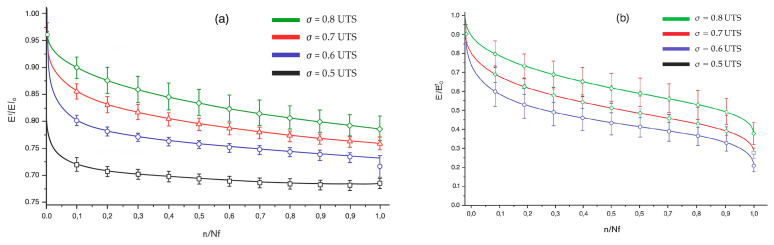
Flax–epoxy composite: Degradation of the elastic modulus at fatigue (concerning the static condition) with the variation in the number of load cycles n for different levels of cyclic load with respect to UTS. (**a**) Unaged specimens and (**b**) post-moisture absorption specimens [48].

**Figure 12:**
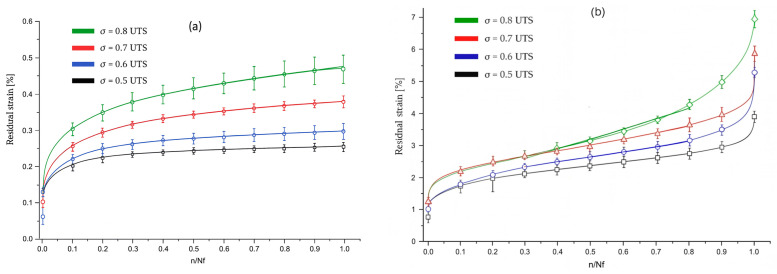
Flax–epoxy composite: Increase in residual strain % at fatigue with varying number of load cycles n for different levels of cyclic load compared to UTS. (**a**) Unaged specimens and (**b**) post-moisture absorption specimens [48].

From the graphs in Figure 11 and Figure 12, the following emerges:

For fatigue elastic modulus (Ef) in unaged samples, the maximum decrease ΔE_fMAX is 25–30% for loads of 0.5 UTS, whether in aged samples, ΔE_fMAX reaches 65–70% for loads of 0.8 UTS.

The difference is attributed to the plasticization of the matrix in aged samples, which allows them to withstand higher loads for longer before failure. On the other hand, in unaged samples, high loads result in rapid and brittle failure.

For residual strain percentage (εr) in unaged samples, Δε_rMAX is approximately 0.46% for loads of 0.8 UTS; in aged samples, Δε_rMAX rises to approximately 6.85% for the same loads.

This indicates that moisture significantly increases the plasticization of the matrix, amplifying residual strain and the accumulation of damage, cycle by cycle.

Finally, in aged samples, the plasticization process continues during fatigue tests, contributing to damage propagation until exponential failure occurs at the end of the material’s useful life (n → Nf).

In addition to the degradation of elastic modulus and residual fatigue strain related to moisture absorption, a crucial aspect in studying the fatigue life of composites is the progressive loss of strength with increasing load cycles. This degradation is more pronounced in materials aged due to moisture absorption than unexposed materials, with the rate of decrease in fatigue stress rising with higher levels of absorbed moisture.

In a 2023 study conducted by Batista et al. [69], specimens of epoxy resin matrix reinforced with sisal fibers (30% by volume) were analyzed in four configurations:

- NaOH-treated fibers, unidirectional (UD) arrangement.
- NaOH-treated fibers, cross-ply (CP) arrangement.
- Untreated fibers, UD arrangement.
- Untreated fibers, CP arrangement.

For each configuration, two sets of specimens were prepared: unaged samples and samples aged by immersion in distilled water at 50 °C for 600 h, following ASTM D 570–22 standards [45].

Moisture absorption at saturation was measured as follows:

- Pure epoxy resin: maximum absorption of 3%.
- Untreated sisal (UD)/epoxy: approximately 20%.
- Untreated sisal (CP)/epoxy: approximately 17.2%.
- NaOH-treated sisal (UD)/epoxy: approximately 11.7%.
- NaOH-treated sisal (CP)/epoxy: approximately 10.0%.

NaOH treatments reduce voids within the fibers, limiting water ingress. Additionally, cross-ply composites absorb less water than unidirectional ones because the fiber architecture mechanically delays water penetration.

Fatigue Stress Analysis:

Results for aged and unaged specimens across all configurations were compared.

The data show a significant reduction in maximum fatigue stress for aged specimens compared to unaged ones, confirming the critical role of moisture absorption in the degradation of mechanical properties in composites.

The study highlighted the fatigue behavior of various materials, showing how moisture absorption affects mechanical properties during load cycles. The results are presented in Table 9.

Aging due to moisture absorption amplifies the formation of microcracks in the matrix, which is caused by the swelling of natural fibers, further weakening the structure. Although moisture absorption negatively impacts the material for NaOH treatment, fiber treatment significantly improves the fiber–matrix interface, ensuring greater mechanical stability compared to untreated samples.

Regarding cross-ply samples, they show higher property loss. This behavior is linked to the 90° fiber arrangement, which promotes crack formation in the matrix, later propagating towards 0° fibers and leading to failure. Again, the treatment significantly enhances performance, reducing the impact of moisture absorption; however, due to the presence of 90° fibers, cross-ply composites are overall less resistant than unidirectional composites.

These findings underline the importance of fiber treatment and material configuration in fatigue resistance, emphasizing how moisture absorption and fiber arrangement significantly influence the mechanical behavior of reinforced composites.

Unidirectional (UD) composites exhibit higher fatigue resistance than cross-ply composites, even under moisture aging conditions. Alkaline treatment of natural fibers enhances overall resistance but increases the fatigue degradation rate due to the greater vulnerability of treated fibers to moisture. In contrast, untreated composites show slower degradation after aging, attributed to matrix relaxation, which can improve their performance under certain conditions.

Finally, post-drying of the specimens eliminates the benefits of matrix relaxation and intensifies moisture-induced damage, making the loss of mechanical properties irreversible. These aspects highlight the importance of moisture management in optimizing the fatigue performance of natural composites.

The 2020 study by Barbière et al. [70] analyzed epoxy resin composites reinforced with hemp fibers (±45°) to validate a fatigue resistance model under moisture absorption conditions. Tests were conducted on specimens conditioned in three ways: Ambient: 21 °C, 48% RH, Wet: Immersed in water at 21 °C for 90–100 h, and Wet/Dry: Post-dried at 40 °C for 48 h.

Tensile Test results:

- Ambient-conditioned specimens displayed the highest tensile strength (σ) and elastic modulus (E) values.
- Wet-conditioned specimens recorded the lowest values (−11% for σ, −36% for E) and exhibited double the failure strain compared to Ambient specimens, attributed to the plasticizing effect of water.
- Wet/Dry specimens showed no change in tensile strength compared to Wet specimens but exhibited partial recovery of stiffness and failure strain due to the elimination of the plasticizing effect.
- Fatigue test results:

(Frequency: 1 Hz and stress ratio R = 0.01)

The Epaarachchi model [67] was used to predict fatigue life:

(5) $$N_{f}={\left[1+\left(\frac{\sigma_{u}}{\sigma_{MAX}}-1\right)\frac{f^{\beta }}{\alpha {\left(1-R\right)}^{1.6-R\left|sin\theta \right|}}{\left(\frac{\sigma_{u}}{\sigma_{MAX}}\right)}^{0.6-R\left|sin\theta \right|}\;\right]}^{\frac{1}{\beta }}$$

where

*N_f_*: Number of fatigue cycles to failure.
*σ_MAX_*: Maximum stress.
*σ_u_*: Ultimate tensile strength (UTS).
*f*: Fatigue test frequency (1 Hz in this study).
*R*: Stress ratio (0.01 in this study).
*θ*: Smallest angle between the load axis and fiber direction (45° in this study).
*α* and *β:* Two parameters to be determined experimentally.

The Wet/Dry and Ambient specimens showed similar parameters (*α* and *β*), indicating comparable fatigue sensitivity. However, the 10% reduction in *σ_u_* for Wet/Dry specimens resulted in shorter fatigue life. In contrast, Wet specimens exhibited different *α* and *β* values and, surprisingly, higher fatigue life under low maximum stress conditions than the other conditioning methods.

Wet/Dry specimens combine the fatigue resistance of Ambient specimens with the permanent damage of Wet specimens behaving like pre-damaged samples with the most significant degradation at the fiber–matrix interface.

## 8. Discussion

Optimizing fiber treatments remains a cornerstone of advancing the performance of natural fiber composites. While NaOH treatments are widely recognized for improving fiber–matrix adhesion, their tendency to increase the material’s sensitivity to moisture highlights the need for alternative approaches. Emerging techniques such as enzymatic treatments, which offer targeted modifications, or the application of nano-coatings, which provide a protective barrier against environmental factors, represent promising directions. Similarly, the refinement of physical surface treatments, despite their high costs, could make these methods economically viable and scalable for industrial applications. A synergistic approach combining chemical, thermal, and physical treatments might also lead to tailored solutions for specific industries, from automotive to construction.

Incorporating natural fibers with biodegradable polymers, such as polylactic acid (PLA) or polyhydroxyalkanoates (PHA), hold significant potential for producing fully sustainable composite materials. These combinations could meet the growing demand for environmentally friendly alternatives, particularly in sectors aiming to reduce carbon footprints. Furthermore, developing advanced polymer matrices with self-healing or hydrophobic properties could mitigate the effects of moisture ingress, enhancing durability and extending service life. Comparative studies of fatigue resistance between conventional petrochemical-based matrices and bio-based alternatives are crucial to identify trade-offs and opportunities for performance improvements.

Additional recent analyses also stress the need for standardized protocols and long-term durability studies, as outlined in the latest comprehensive reviews.

Despite advancements, significant gaps remain in understanding long-term performance under environmental stresses. Comprehensive studies on moisture absorption, temperature fluctuations, and their combined effects with mechanical loading are still limited. Expanding research into challenging environments, such as tropical climates or marine conditions, could shed light on durability issues and guide the development of more resilient composites. Understanding fatigue behavior under thermomechanical conditions where simultaneous thermal and mechanical stresses are applied is vital for assessing real-world applicability in aerospace or offshore engineering sectors.

Advances in predictive modeling are equally critical for the next generation of composite materials. Enhanced models that account for complex phenomena like fiber swelling, interface degradation, and microdamage evolution could provide more accurate forecasts of material behavior. Non-destructive techniques such as X-ray microtomography and advanced digital imaging systems offer invaluable data for refining these models. Integrating these datasets with machine learning algorithms could revolutionize material design, enabling researchers to link microstructural properties with macro-level performance efficiently.

Interdisciplinary collaborations between materials science, computational modeling, and industrial applications will be essential. Integrating sustainable practices, cost-effective manufacturing processes, and innovative treatments will drive the next wave of breakthroughs in natural fiber composites. By addressing these challenges and opportunities, researchers can unlock the full potential of these materials, paving the way for a future where high performance is achieved without compromising sustainability.

## 9. Conclusions and Future Directions

This review has provided a comprehensive overview of how moisture absorption affects the mechanical and fatigue properties of natural fiber composites (NFCs), with particular attention to the influence of fiber type, surface treatments, environmental conditions, and composite architecture. The analysis confirms that NFCs exhibit substantial potential for sustainable structural applications, but also face significant challenges related to their hydrophilic nature and sensitivity to environmental exposure.

Key findings include:

Moisture absorption leads to notable reductions in tensile strength and elastic modulus, especially in composites with high fiber content or poor fiber–matrix adhesion.

Fatigue resistance is strongly affected by humidity and water uptake, with moisture-induced plasticization lowering stiffness and accelerating failure mechanisms.

Surface treatments, particularly alkaline and silane, can significantly improve both mechanical properties and resistance to moisture, though their effectiveness depends on treatment concentration and process conditions.

Hybridization with synthetic fibers and optimized fiber orientation have shown to enhance fatigue life and reduce moisture sensitivity.

Despite the volume of experimental data available, several limitations remain, as follows:

- A lack of standardized testing protocols makes it difficult to compare results across studies.
- Long-term durability under cyclic environmental conditions (e.g., freeze–thaw and marine immersion) remains insufficiently investigated.
- Many studies focus on quasi-static or short-term behavior with limited insight into aging effects over service lifetimes.

Future research should focus on the following:

- Developing predictive models that couple moisture diffusion with mechanical degradation over time.
- Exploring novel surface treatments or bio-based coupling agents that enhance compatibility without compromising sustainability.
- Investigating multiscale damage mechanisms through in situ monitoring techniques (e.g., acoustic emission and micro-CT) under realistic environmental and mechanical loads.
- Establishing standardized test protocols to enhance reproducibility and enable comprehensive meta-analyses of moisture-aging and fatigue behaviors across NFC systems.

By adopting a multidisciplinary approach that integrates materials science, mechanical engineering, and environmental modeling, the reliability and practical applicability of natural fiber composites can be substantially improved. Addressing these challenges will accelerate the development of next-generation bio-based materials capable of delivering high performance in demanding structural applications, while also supporting global sustainability goals.

## Figures and Tables

**Table 1:** Comparative mechanical properties of E-glass composites and natural fiber hybrid composites including experimental tensile and displacement data [3].

Sample	Fiber Volume (%)	Young’s Modulus (GPa)	Tensile Stress (MPa)	Tensile Strength (kN)	Max Displacement (mm)	Elongation (%)	Young’s Modulus (MPa, test)	Testing Standard(s)	Testing Equipment
E-Glass Composites	54 (mean)	40 (mean)	1014 (mean)	–	–	–	–	ASTM D3039 [5] (tensile)	Universal Testing Machine with extensometer
Flax Composites	43 (mean)	25 (mean)	255 (mean)	–	–	–	–	ASTM D3039 (tensile)	Universal Testing Machine with extensometer
GFRP + Abaca	–	–	44.5	5.825	8.56	15.05	270	ASTM D3039 (tensile), ASTM D7264 [6] (flexural)	Universal Testing Machine with extensometer
GFRP + Jute	–	–	46.5	6.23	9.12	15.72	250	ASTM D3039 (tensile), ASTM D7264 (flexural)	Universal Testing Machine with extensometer
GFRP + Abaca + Jute	–	–	57.0	7.1075	10.0	18.18	290	ASTM D3039 (tensile), ASTM D7264 (flexural)	Universal Testing Machine with extensometer

**Table 2:** Mechanical properties and fatigue performance of pure epoxy and fiber reinforced composites with 15% and 45% fiber volume fractions (Vf).

Property/Stress (MPa)	Pure Epoxy	15% Vf Composite	45% Vf Composite
UTS (MPa)	36.56	57.95	100.56
Young’s Modulus (GPa)	2.92	3.96	7.78
32.9 [MPa] (Fatigue cycles)	6	55	300
29.3 [MPa] (Fatigue cycles)	18	44	4247
25.6 [MPa] (Fatigue cycles)	120	1209	63,110
22.0 [MPa] (Fatigue cycles)	200	78,950	113,521
18.3 [MPa] (Fatigue cycles)	686	550,010	758,963

**Table 3:** Results on the mechanical properties of alkaline and silane treatments for different natural composites. (UTS = tensile strength, ST = shear strength, FT = flexural strength, D = hardness, and E = Young’s modulus) [35].

Fiber Reinforced Composites	Treatment Applied	Properties Achieved
Abaca reinforced composites	Alkali treatment with NaOH 5%	UTS: ~+8% ST: +32%
Coconut fiber reinforced polymer composites	Alkali treatment with NaOH 5% at 20 °C for 30 min	UTS: +17.8% FT: +16.7%
Benzoxazine resin reinforced with alfa fibers	Alkali treatment with NaOH 5% for 5 h	D: +29% FT: +37% E: +10%
Oil palm/bagasse fiber-reinforced phenolic hybrid composites	Silane treatment with 2% + 4% H_2_O_2_	UTS: +56% FT: +120%
Acacia tortilis fiber-reinforced natural composite	Alkali treatment with NaOH 10%	UTS: +27.5%
Flax/banana/industrial waste tea leaf fiber-reinforced hybrid polymer composite	Alkali treatment with NaOH 5% for 12 h at 27 °C	UTS: +7% FT: +5%
Mutingia Calabura bark fiber-reinforced green epoxy composite	Alkali treatment with NaOH 5%. Silane treatment	UTS: +37.75% NaOH UTS: +28.92% Silani

**Table 4:** Fatigue data for epoxy and polyester composites at different stress levels under stress-to-tension cyclic loading (R = 0.1). Comparison of NaOH treated and untreated sisal fibers.

Resin	Fiber Type	MAX Stress (%)	σ_MAX_ (MPa)	Frequency (Hz)	Mean Failure Cycles (N)
POLYESTER	Not treated	75	167	2.7	2710
		60	133	3.3	69,757
		50	111	4	544,296
		35	78	5.7	3,811,072
	Treated	75	245	2.1	15
		60	172	2.6	6956
		50	143	3.1	273,792
		35	100	4.4	3,394,240
EPOXY	Not treated	90	297	1.5	0.5
		85	280	1.6	0.5
		80	264	1.7	2
		75	247	1.8	1128
		65	214	2.1	35,075
		60	198	2.2	21,604
		50	165	2.7	104,720
		35	115	3.9	2,372,300
	Treated	90	302	1.5	10
		85	285	1.6	287
		80	268	1.7	853
		75	252	1.8	4135
		70	235	1.9	8407
		65	218	2	37,756
		60	201	2.2	51,257
		50	168	2.6	149,398
		35	117	3.8	1,239,806

**Table 5:** Fatigue life data for epoxy resin composites at different stress levels under tensile–compressive load (R = −1). Comparison of NaOH treated and untreated fibers.

	Fiber Type	MAX Stress (%)	σ_MAX_ (MPa)	Frequency (Hz)	Mean Failure Cycles (N)
EPOXY RESIN	Not treated	95	99	2.0	268
		90	94	2.1	352
		80	83	2.4	793
		75	78	2.6	2757
		65	68	3.0	92,631
		60	62	3.2	159,562
		50	52	3.8	359,181
		35	42	4.8	1,018,278
	Treated	90	110	1.8	92
		75	92	2.2	443
		70	86	2.3	775
		65	80	2.5	15,624
		60	74	2.7	27,264
		50	61	3.3	145,227
		35	43	4.7	1,000,000

**Table 6:** Percentage reduction in mechanical properties for glass–hemp hybrid composites.

Fiber Percentage (%)	Reduction in Tensile Strength (%)	Reduction in Young’s Modulus (%)
0% (pure PLA)	0.11	8.48
15%	11.2	9.05
20%	13.9	16.7
25%	16.7	20.5

**Table 7:** Absorption % at saturation and diffusion coefficient for different fiber types and immersion temperatures [59].

Sample	Temperature (K)	Saturation WA (%)	Diffusion Coefficient (10^−5^ cm^2^ × s^−1^)
Bow Hemp	300	62	0.52
	310	64	1.11
	320	67	1.28
	330	76	1.32
	340	80	1.36
Okra	300	64	0.54
	310	66	1.14
	320	69	1.30
	330	78	1.34
	340	81	1.38
Betel Nut	300	38	0.28
	310	51	0.68
	320	58	1.04
	330	66	1.15
	340	70	1.19

**Table 8:** Diffusivity coefficient [m^2^/s × 10^13^] for flax/PP pulp composites calculated after immersion in water at 30, 50, 70, and 100 °C for 7 days.

Coupling Agent	Reinforcement %	Diffusion Coefficient at Different T (°C)			
		30	50	70	100
MAPP1	2	0.516	5.9	6.37	18.86
	4	0.354	5.39	4.25	15.50
	7	0.239	5.45	4.28	16.43
	10	0.263	1.95	8.51	19.18
	15	0.291	5.87	7.88	19.99
MAPP2	2	0.479	4.59	7.65	23.12
	4	0.408	5.60	7.71	25.97
	7	0.351	4.27	7.34	28.88
	10	0.352	6.52	8.93	25.29
	15	0.355	6.92	7.12	25.98

**Table 9:** Loss in tensile strength of sisal/epoxy composites after 50,000 and 100,000 loading cycles. Comparison between architectures: UD (untreated unidirectional), NaOH UD (NaOH-treated unidirectional), CP (untreated cross-ply), and NaOH CP (NaOH-treated cross-ply).

	Tensile Strength Reduction (%)			
	Unaged Samples		Aged Samples	
	50,000 Cycles	100,000 Cycles	50,000 Cycles	100,000 Cycles
UD	10.1	20.8	13.7	18.2
NaOH UD	4	6	8	12
CP	17.9	29.3	14.1	22.9
NaOH CP	9.3	12.1	9.4	13

## Data Availability

No new data were generated in this study. The data used are from previously published sources, as cited in the article.

## Abbreviations

The following abbreviations are used in this manuscript:

Abbreviation	Definition
NFCs	Natural Fiber Composites
D	Hardness
D	Diffusion Coefficient, Measures Rate of Water Penetration in a Composite
E	Young’s Modulus
E-Glass	Type of Glass Fiber
EMC	Equilibrium moisture content
FT	Flexural Strength
HDPE	High-Density Polyethylene
LDPE	Low-Density Polyethylene
M∞	Saturation Moisture Content
MAPE	Maleated Polyethylene (Coupling Agent)
PFC	Polymer Fiber Composite
PLA	Polylactic Acid
PP	Polypropylene
R Ratio	Load Ratio in Fatigue Testing (Minimum Load/Maximum Load)
SEM	Scanning Electron Microscopy
S–N Curve	StresS–Number of Cycles Curve (Fatigue Life Representation)
ST	Shear Strength
Tg	Glass Transition Temperature
UTS	Ultimate Tensile Strength
V_f_	Fiber Volume Fraction
WA	Water Absorption
η	Loss Factor (Fatigue Analysis)
σ_MAX_	Maximum Stress
